# Progress towards elimination of onchocerciasis in the *Region du Sud-Ouest* of Burkina Faso which was previously subject to a recrudescence event after vector control

**DOI:** 10.1371/journal.pntd.0012118

**Published:** 2024-04-29

**Authors:** Achille Sindimbasba Nikièma, Lassane Koala, Rory J. Post, Appolinaire Kima, Justin Compaoré, Claude M. Kafando, Jean Baptiste Nana, Clarisse Bougouma, Babacar Faye, Soungalo Traoré, Roch Kounbobr Dabiré

**Affiliations:** 1 Ministère de l’Enseignement Supérieur, de la Recherche et de l’Innovation, Institut de Recherche en Sciences de la Santé, Direction Régionale de l’Ouest (IRSS/Bobo-Dioulasso), Ouagadougou, Burkina Faso; 2 Université Cheikh Anta Diop, Dakar, Sénégal; 3 School of Biological & Environmental Sciences, Liverpool John Moores University, Liverpool, United Kingdom; 4 Ministère de la Santé, Direction de la Protection de la Santé de la Population, Programme National de Lutte Contre les Maladies Tropicales Négligées, Ouagadougou, Burkina Faso; Washington University School of Medicine, UNITED STATES

## Abstract

**Background:**

The *Sud-Ouest* region of Burkina Faso (especially the Bougouriba valley) has been historically problematic with respect to onchocerciasis control, with a recrudescence of infections after vector control carried out the WHO Onchocerciasis Control Programme was halted in 1989. After 1996, mass drug administration of ivermectin was instigated to control the recrudescence so that it would no longer constitute a public health problem. However, in 2010 WHO changed its recommended policy from control to elimination, and in 2013 biannual Community-Directed Treatment with Ivermectin (CDTI) was instigated. Epidemiological surveys were carried-out in 2011 and 2018 to determine whether CDTI was producing a decline in infection levels and progress towards elimination.

**Methodology/Principal findings:**

A cross-sectional study was conducted across 20 villages in four health districts in 2011 and 29 villages in 2018. Individuals aged five years and above were examined by skin-snip, and the prevalence and microfilarial load was determined for each village.

In 2011, 75% of villages had some infections and 20% had prevalences >5%, with a mean prevalence across all villages of 2.63% (range 0.0–9.7%), and community microfilarial load ranging from 0 to 0.25 microfilariae per biopsy. In 2018, nine villages (= 31% of total) had some infections, with prevalences ranging from 0.41% to 3.54%, and a mean prevalence across all villages of 0.37%. Community microfilarial load ranged from 0 to 0.1. Amongst those people found to be microfilarial positive, 87% had a history of migration.

**Conclusions/significance:**

The endemicity of onchocerciasis infection in the *Sud-Ouest* region has declined to low levels and seems to be progressing towards elimination. Our findings indicated that biannual CDTI is having good effect, but it should continue for a number of years to ensure elimination of transmission. However, progress towards elimination has a troublesome history in this region, and it would be advisable to select more sentinel villages to have confidence in any future epidemiological and entomological surveys, especially Stop-MDA surveys. With positive individuals migrating between countries, cross-border collaboration needs more attention to ensure effective treatment for onchocerciasis elimination.

## Author summary

Onchocerciasis is a disease caused by infection with the parasitic nematode, *Onchocerca volvulus*, which is transmitted by blood-sucking blackflies, causing blindness, skin disease and epilepsy. The World Health Organization has listed the disease for elimination using the drug ivermectin, but the density of surveillance points necessary to ensure that elimination has been achieved throughout an area is not well understood.

Most of Burkina Faso became free from onchocerciasis after the regular application of insecticides to the blackfly breeding sites from 1975 to 1989. However, the South-West Region suffered a speedy recrudescence, probably because an insufficient number of monitoring sites were not representative of the situation throughout the whole area and blackfly control was stopped prematurely. Mass distribution of ivermectin was introduced in 1996 to control the recrudescence and CDTI in 2013 to eliminate transmission. The objective of this study was to carry out epidemiological surveys in 2011 and 2018 to see if ivermectin is reducing human infections and eliminating the recrudescence.

In 2011, 75% of villages still had some infections and the mean number of people infected across all villages was 2.63%. However, in 2018, only 31% of villages had any infections, with mean numbers infected across all villages of 0.37% showing that there is good progress towards elimination.

## Introduction

Onchocerciasis is a vector-borne neglected tropical disease caused by infection with *Onchocerca volvulus*, a human parasite with no known animal reservoir [1]. In West Africa, the parasite is transmitted from human to humans by blood-sucking female blackflies of the *Similium damnosum* complex, which breed in fast-flowing streams and rivers [1,2]. More than 217.5 million people live in areas at-risk of onchocerciasis [3], and in Africa it is endemic in 31 countries, but the World Health Organisation (WHO) has targeted it for global elimination with 31% of countries achieving verification of elimination by 2030 [4]. Onchocerciasis is a rural disease which is disfiguring and disabling because it causes skin disease and visual impairment including significant levels of blindness, and recent studies have also associated onchocerciasis with epilepsy [5,6]. The adult female worm is found in subcutaneous nodules, where it releases larvae (microfilariae) which invade the skin and the eyes. The pathogenesis of onchocerciasis is due to the microfilariae which cause damage to the skin and eye tissues when they die.

Until 1987, there were no safe drugs which could be used to treat onchocerciasis, and prevention of infections was by larviciding the vector breeding sites to reduce the numbers of biting vectors. However, in 1987 ivermectin was introduced for use against onchocerciasis, and it remains the only drug recommended for treatment [7]. It has very few side effects, and it is therefore safe and easy to use in mass treatment campaigns (except in areas of co-endemicity with loiasis–which does not occur in Burkina Faso) [8]. Ivermectin kills microfilariae and thus reduces the microfilarial loads of treated communities, and whilst it has little macrofilaricidal effect, it temporarily sterilizes the adult female worm, which will usually recover reproduction a few months after treatment [9]. Thus, ivermectin is routinely administered annually or biannually to at-risk communities in endemic areas of Africa in order to remove microfilariae from the skin and consequently to interrupt transmission of onchocerciasis [10].

Given the safe and easy use of ivermectin in mass treatment campaigns, and because of its availability as a donation ‘for as long as it is needed’ by the manufacturer Merck & Co Inc, the World Health Organization developed a sustainable strategy for Mass Drug Administration called Community Directed Treatment with Ivermectin (CDTI) because it involves the endemic community in decision-making and drug distribution to control onchocerciasis [11]. This strategy became the mainstay of onchocerciasis control in the countries participating in the WHO African Programme for Onchocerciasis Control (APOC) [2,12,13,14] which aimed to eliminate onchocerciasis as a public health problem throughout Africa, but not necessarily to eliminate transmission, which was considered to be impossible with the tools available at the time [15]. This meant that ivermectin might have to be distributed indefinitely, and this would probably be unsustainable, but after a few years the progress towards elimination using ivermectin in south/central America and the proof of the feasibility of elimination of onchocerciasis with ivermectin in Africa, led APOC to shift from control to elimination [9,16,17,18]. This required CDTI to be extended into areas with transmission which had previously not been subject to CDTI because they had low prevalence and onchocerciasis was not considered to be of public health importance. The new policy of elimination of transmission (which would result in elimination of the parasite) required such areas to be included in the programme. The reproductive period of the mature adult female lasts between nine to eleven years, (during which, the mature female produced on average 1,000 to 2,000 microfilariae per day) [19,20], and therefore CDTI would have to continue for at least nine years with high levels of therapeutic and geographic coverage.

In Burkina Faso, onchocerciasis control was first applied using vector control (larviciding the vector breeding sites with DDT) in 1962 to 1963 in the headwaters of the Mouhoun (= Black Volta), Comoé and Farako rivers, but not in *Region Sud-Ouest* (= South-West administrative Region) [21], and this was followed in 1969 by vector control (again, larviciding with DDT) throughout the Comoé river and its tributaries in Burkina Faso, but *Region Sud-Ouest* was again not included [22]. However, this campaign led directly on to Phase One of the WHO Onchocerciasis Control Programme (WHO-OCP) in February 1975, which carried out weekly larviciding (using temephos) over a very wide area, including all the rivers within the *Region Sud-Ouest* (including the Mouhoun river where it forms the border with Ghana) [23], and continuing for more or less 15 years. The *Region Sud-Ouest* had high levels of onchocerciasis, and surveys carried out before 1975 indicated that onchocerciasis was hyperendemic with a prevalence of 83.7% in some places, a community microfilarial load (CMFL) of 98 mf/s, a blindness prevalence of 11.6%, an annual vector biting rate (ABR) of more than 6000, and an annual transmission potential (ATP) >600 [24]. Larviciding stopped in some tributaries of the Bougouriba river (including the whole of the Naimo river) in 1987, and in late 1989 larviciding was discontinued throughout WHO-OCP Phase One area (including the whole of *Region Sud-Ouest*) because onchocerciasis had been reduced to levels which were considered insignificant and no longer constituted a public health problem.

By 2001 there were the first signs of a resurgence of infections in the Comoé river valley in *Region des Cascades* (immediately east of *Region du Sud-Ouest*) and this was also indicated by subsequent skin-snip surveys in 2007 and 2008 and finally confirmed in 2010 [25]. After a final survey in 2011, CDTI was implemented in July 2011, for the first time anywhere in Burkina Faso, with the objective to control/eliminate onchocerciasis recrudescence along the Comoé river valley. It was administered biannually to quickly decrease the prevalence which had reached over 70% in some places [25].

The recrudescence of onchocerciasis in the *Cascades* region raised questions about the epidemiological situation in the other river basins, which had previously been controlled by WHO-OCP in Burkina Faso. To investigate this situation the National Onchocerciasis Control Programme conducted epidemiological surveys in the river basins of the *Boucle de Mouhoun*, *Hauts-Bassins*, *Centre-Sud* and *Centre-Est* regions in 2014, and these surveys showed that onchocerciasis was not recrudescent, with prevalences recorded below 5%, and the few positive people were almost all immigrants from Côte d’Ivoire [26]. However, the situation in the *Sud-Ouest* region was already known to be problematic. During 1976–1999, the organized establishment of farming communities in the valleys, which were thought to be safe from onchocerciasis, had led to environmental modifications which created artificial vector breeding sites which were not recognized, and in 1992, entomological data collected at Batié on the Bambassou river gave first indications of a deteriorating situation. In 1994, children newly infected since 1992 were found with an incidence > 1.8% in some villages. Even in the areas where in 1991 a low infectivity rate (0.6%) was found, an increase to 5.9% was observed in 1995 [24]. As a result, MDA using ivermectin was introduced 3-times per year, at first carried out by mobile health teams (1996–1999) and later by community-based distribution (1999–2013), and some ground-based larviciding was carried out near one village in 1997–1998. Therapeutic coverage for MDA was quite good (70–80%), but geographic coverage was problematic, because it was unclear how many villages needed to be treated. Furthermore, after an initial strong effort to get a good coverage, the number of villages treated dropped from around 180 per year to below 100 per year after 1998 [24]. A skin-snip survey carried out in 2011 showed that onchocerciasis was still a public health problem (see Results below) and to be able to progress towards elimination, the National Onchocerciasis Control Programme introduced biannual CDTI in May 2013, and the *Sud-Ouest* region became the second endemic area in Burkina Faso to implement CDTI. Annual treatment coverage rates were above 65% of the total population and geographical coverage rates were 100% from 2013 to 2018. WHO-APOC recommended that surveys to measure the trend in prevalence and intensity of infection should be carried out after six years of treatment [2], and hence the national programme for neglected tropical diseases control carried out epidemiological surveillance of onchocerciasis in selected villages in the *Sud-Ouest* region in 2018. (Table 1)

**Table 1:** Summary history of the main events in the control of onchocerciasis in *Region Sud-Ouest* of Burkina Faso.

Year	Event
1975	Weekly larviciding by WHO-OCP commences in February 1975
1989	Larviciding by WHO-OCP discontinued at end of 1989
1992	First signs of a recrudescence in *Region Sud-Ouest* from routine entomological survey
1994/95	Recrudescence confirmed by skin-snip surveys
1996–1999	3 x p.a. mass drug administration (MDA) with ivermectin introduced using mobile teams
1999–2013	Mobile teams replaced by Community Based Treatment with Ivermectin
2010	WHO-APOC changes its recommended strategy from control to elimination
2011	Epidemiological survey by skin-snip shows that oncho is still a public health problem
2013-present	Biannual CDTI
2018	Epidemiological Survey by skin snip to assess progress towards elimination

The objective of our study was to determine the status of onchocerciasis prevalence and Community Microfilarial Load (CMFL) in 2018 after five consecutive years of biannual CDTI, and to assess progress towards elimination

## Materials and methods

### Ethical considerations

The National Onchocerciasis Control Programme received blanket approval from the Ethics Committee of the Ministry of Health in 2009 for carrying out the routine activities for the control and elimination of onchocerciasis (including epidemiological evaluation by skin-biopsy as carried out for this study). The protocol for diagnosis used is that previously used by WHO-OCP and WHO-APOC for onchocerciasis epidemiological survey. In each community, the day of the survey, the purpose and method of the examination was explained to the inhabitants who were present. Those who expressed their consent to participate in the study were examined. Participants aged 18 years and over gave their verbal consent and for those under 18 years of age, their parents or guardians verbal consent was obtained before examination. The National Programme keeps the data confidential at the Ministry of Health with access restricted to the data manager and the programme manager.

### Study area

**Fig 1:**
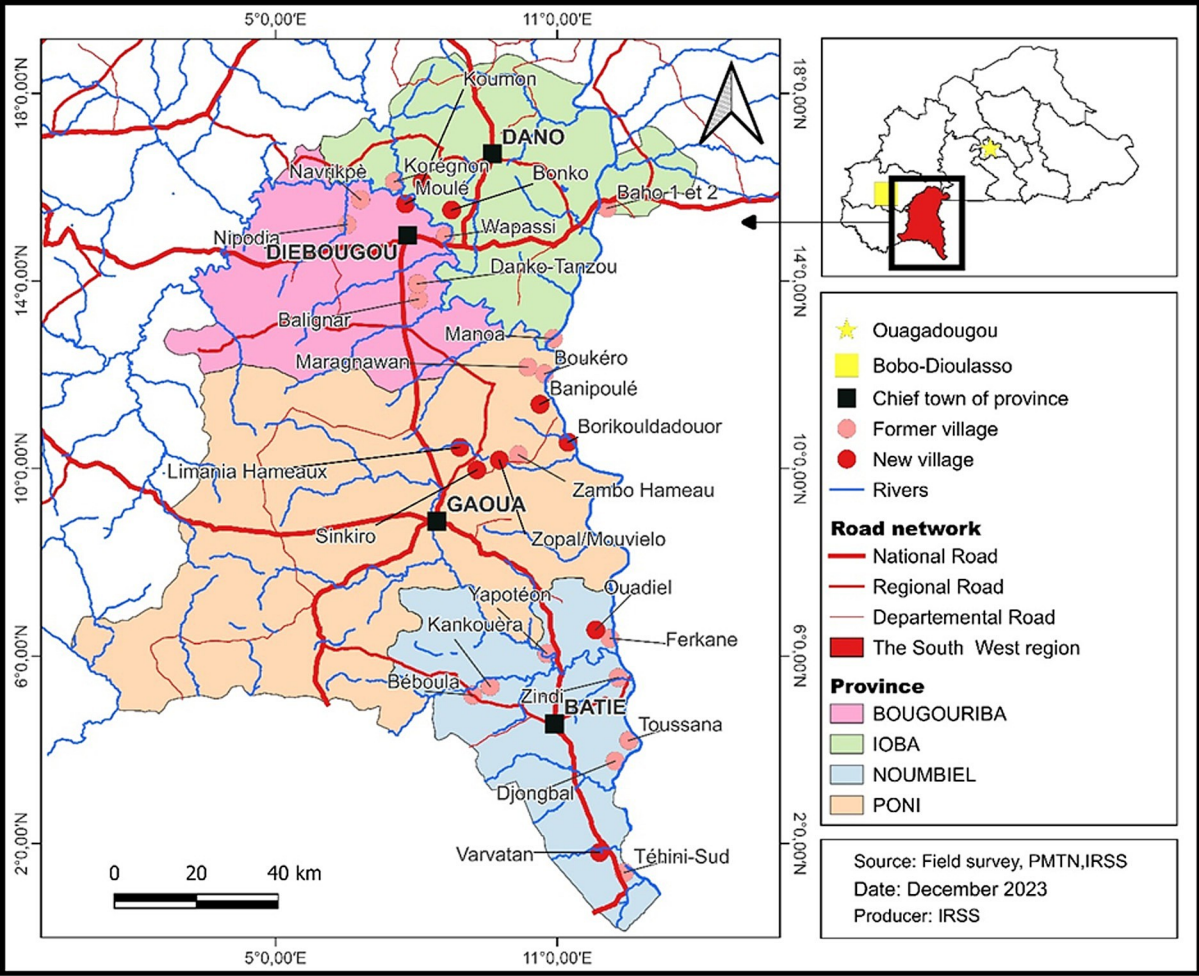
Location of the 2018 survey sites in the four health districts in the *Region Sud-Ouest* of Burkina Faso. **Notes:** The Bougouriba River is the river just north of Diebougou town and forms most of the boundary between Bougouriba and Dano Provinces. The river flows more or less eastwards through the study area and eventually joins the Mouhoun River (sometimes known as the Black Volta) which forms the international border with Ghana along the eastern border of the *Region Sud-West*. The Bougouriba valley consists of the area drained by the Bougouriba River and all of its tributaries. Fig 1 was constructed by PMTN (Programme national de lutte contre les Maladies Tropicales Négligées which is part of the Direction Générale de la Santé et de l’Hygiène Publique) in conjunction with IRSS (Institut de Recherche en Sciences de la Santé). The basemap was obtained from the Base Nationale des Données Topographique (BNDT) of the l’Institut Géographique du Burkina (IGB), and is provided freely to other government departments.

This study was carried out in the *Sud-Ouest* health region, in the south-western part of Burkina Faso (Fig 1). The region shares its southern borders with two neighboring countries, Côte d’Ivoire and Ghana. It is bordered to the northeast by the *Centre-Ouest* region, to the north by the *Boucle du Mouhoun* region, and to the west by the *Hauts-Bassins* and *Cascades* regions. It is subject to significant population movements from inside of the country in search of fertile land, pasture and gold, as well as cross-border movements, mainly from Côte d’Ivoire and Ghana, for plantation work and trading. The relief is very rugged with hills and lowlands, and the vegetation is of the southern sudan savannah type (also known as sudano-guinea savannah) with gallery forests along the rivers. The annual rainfall is between 900 and 1200 millimeters with a rainy season that lasts from April to October and a dry season for the rest of the year. The hydrographic network belongs mainly to the Mouhoun river basin (= Black Volta) which forms the eastern border of the whole study area, with a large number of tributaries. The Bougouriba river is of particular importance to this study and the part of the Bougouriba valley included in this study includes 12 villages shown on Fig 1. The vectors which have been identified from the region have all been *S*. *damnosum* s.str. and *S*. *sirbanum* (with *S*. *sirbanum* dominant) [27].

### Selection of villages

Previous studies had used only 19 sentinel villages, but to obtain better coverage, the 2011 and 2018 surveys examined a total of 20 and 29 villages respectively from the four health districts: Batié (8 and 10 villages respectively), Gaoua (2 and 4 villages respectively), Diébougou (7 and 9 villages respectively) and Dano (3 and 6 villages respectively). This represented a 53% increase (from 19 to 29) in the number of sentinel villages (Fig 1). Eighteen of these villages were surveyed in both 2011 and 2018. Before the cessation of vector control and the subsequent recrudescence there had been only six sentinel villages for epidemiological assessments in the Bougouriba valley, and to obtain an improved coverage 12 sentinel villages were surveyed in 2018 (Fig 1).

The villages were chosen to give broad coverage across the river basins within the study area, and with respect to WHO-OCP criteria for sentinel sites: (i) having experienced a high level of onchocerciasis infection in the past; (ii) being located near major breeding sites for vector blackflies; (iii) being located ‘on the first line’; (iv) their population being around 200–300 inhabitants; and (v) having a relatively stable population; the migratory movements of village inhabitants being limited in time and space [28,29]. The use of these criteria for selecting villages has been standard practice in monitoring and evaluation surveys (impact assessments) for onchocerciasis control and elimination. The objective is not to make an overall assessment of onchocerciasis across the entire study area, but to serve as a basis for evaluating the treatment actions carried out, and progress towards elimination. For this purpose, villages are chosen because they are expected to have the highest rates of infection and to be the most sensitive indicators of the impact of interventions. The concept of first-line villages was not used exclusively for the selection of survey villages, but it is important because in West Africa (and particularly in the south-western region of Burkina Faso) previous studies have shown that first-line villages that were often the most affected by onchocerciasis and the consequences appeared earlier in these villages than in the second- and third-line villages [30]. Sentinel villages are shown in Fig 1, and associated breeding sites are mapped in S1 Fig.

### Survey method

The epidemiological surveys were carried out in the selected villages between January and February 2011 and November-December 2018, 11 months after the last cycle of ivermectin treatment in both years, which required the suspension of the mid-year round of CDTI in 2018 because the *Sud-Ouest* region was under biannual CDTI at that time. The survey method followed that developed by WHO-OCP and WHO-APOC [29]. The day before the visit to the village by the team from the National Programme for Neglected Tropical Diseases Control, the inhabitants of the village were informed of their imminent arrival by the local health authorities, religious leaders, and the community distributors of ivermectin (CDDs). On the day of the survey, the team was helped by local health professionals (nurses), and first carried out general sensitization of the village inhabitants with respect to onchocerciasis and explained the objectives of the visit and the planned activities. The geographical coordinates of the village were recorded using a Geographical Positioning System (GPS). Then the team conducted a census of the village population, household by household. Lastly, all inhabitants who were present on the day of the survey, aged five years and over and agreed to participate, were examined using the skin-snip method (see below) for onchocerciasis parasitological diagnosis according to the method of Prost and Prod’hon [31].

### Onchocerciasis parasitological diagnosis method

The assessment of infections by direct observation of microfilariae in skin-snips by microscopy is highly specific and has been used successfully for many years to assess the effects of interventions aimed at controlling onchocerciasis [9,31,32]. It is not sufficiently sensitive for the assessment of interruption of transmission [33], but it is still considered suitable for routine impact assessments for following progress towards elimination [34]. The skin-snip method is cheap, highly specific and familiar to both operatives and the public in Burkina Faso, and is directly comparable to historical data, and therefore it was appropriate for this study, using the standard method [31].

A skin biopsy was taken from the left and right iliac crests of each individual using a 2 mm Holth sclerotomic punch, and then each biopsy was incubated in distilled water for 30 minutes after which it was examined under a microscope to count the number of *O*. *volvulus* microfilariae which had emerged. Biopsies which were negative after 30 minutes incubation, were incubated in physiological saline for 24 hours and then re-examined [28,31].

### Migration survey

All individuals who were skin-snip positive were questioned about their migration history over the past ten years using standardized questionnaires [35]. The objective was to determine the possible geographical origin of any infections.

### Data analysis and parasitological indices

The level of infection in a village was determined by prevalence (% of people carrying skin microfilariae) and the Community Microfilarial Load (CMFL). The crude prevalence of infection was determined as the ratio of the number of people with microfilariae to the total number of people examined by skin biopsy. The standardized prevalence is age- and sex-standardized using the OCP reference population. The standardized prevalence was introduced by WHO to enable comparison of villages and longitudinal monitoring of infection trends in sentinel villages in order to correct certain sampling distortions and enable results to be compared over time and space, the indices are adjusted by reference to a standard WHO defined population [28]. The CMFL was the geometric mean number of microfilariae detected in the skin samples of all persons aged 20 years or older examined in each community [36]. Data collected from positive individuals were entered into an excel template and software R version 3.5.3 was used for statistical analyses.

## Results

### 2011 Survey

Results from the 2011 survey are presented in Table 2. Out of the 20 villages surveyed, 75% (n = 15) had some infections and 20% (n = 4) had prevalences >5%, with a mean prevalence across all villages of 2.6% (range 0.0–9.7%), and community microfilarial load ranging from 0 to 0.25 microfilariae per skin-snip (mf/ss). A migration survey was carried out and amongst the 113 positive people, 25 had a history of migration to neighboring countries (Cote d’Ivoire and Ghana) and 88 had been continuously resident in their respective villages.

**Table 2:** Results of the 2011 skin-snip epidemiological survey showing microfilarial prevalence and community microfilarial load (CMFL) in all study villages.

	River Basin	District	Village	Number examined by skin snip	Number infected	Crude % prevalence	Standardised % prevalence	CMFL mfs/ss
**1**	Bambassou	Batié	Béboula	109	0	0	0	0
**2**	Bambassou	Batié	Kankouèra	85	2	2.4	2.4	0.02
**3**	Mouhoun	Batié	Téhini Sud	145	7	4.8	5.3	0.18
**4**	Mouhoun	Batié	Djonbal	279	27	9.7	9	0.25
**5**	Mouhoun	Batié	Toussana	315	27	8.6	10.1	0.25
**6**	Mouhoun	Batié	Ferkane	144	9	6.3	4.2	0.22
**7**	Bambassou	Batié	Yapotéon	212	6	2.8	3.1	0.04
**8**	Mouhoun	Batié	Zindi	225	3	1.3	1.3	0.04
**9**	Mouhoun	Gaoua	Boukéro	287	0	0	0	0
**10**	Mouhoun	Gaoua	Maragnawa	232	5	2.2	1.7	0.04
**11**	Bougouriba	Diébougou	Navrikpe	251	0	0	0	0
**12**	Bougouriba	Diébougou	Nipodja	108	0	0	0	0
**13**	Bougouriba	Diébougou	Balignar	203	7	3.4	2.9	0.07
**14**	Bougouriba	Diébougou	Sikongo	189	1	0.5	0.2	0.03
**15**	Bougouriba	Diébougou	Wapassi	239	1	0.4	0.6	0.07
**16**	Bougouriba	Diébougou	Danko Tanzou	171	10	5.8	5.3	0.14
**17**	Bougouriba	Diébougou	Benkadi	289	3	1.0	1.2	0.04
**18**	Mouhoun	Dano	Manoa	154	3	1.9	1.6	0.03
**19**	Mouhoun	Dano	Baho	91	1	1.1	0.8	0.02
**20**	Bougouriba	Dano	Korégnon	510	1	0.2	0.2	0
**Totals & Means**				**4,238**	**113**	**2.62**	**2.50**	**0.07**

**Notes:** Of the 113 positives, 95 people were over the age of 19, 18 people were less than 19 years old (and of these, 13 people were less than 10 years old).

### 2018 Survey—prevalence and microfilarial load

The number of individuals examined by district is presented in Table 3 and the full data set of results listing individual villages from 2018 is presented in Table 4. In the four health districts (Batié, Gaoua, Diébougou, Dano), a total of 8,522 individuals were included in the census, and skin snips was performed on 5,621 of them. The overall percentage of individual participating in skin snip was 66.0%. Depending on the district, the participation rate varied from 61.0% in Diébougou district to 71.6% in Batié district.

**Table 3:** Numbers villages surveyed in each health district in 2018 and numbers of inhabitants examined.

Health district	Number of villages	Numbers of inhabitants	Number examined	Percentage examined %
Batié	10	3047	2181	71.6
Gaoua	4	898	608	67.7
Diébougou	9	2339	1427	61.0
Dano	6	2238	1405	62.8
Total	29	8522	5621	66.0

**Table 4:** Results of the 2018 skin-snip epidemiological survey showing microfilarial prevalence and community microfilarial load (CMFL) in all study villages.

Health District	Village	River basin	Census population	No. people examined	No. Positive	Crude prevalence %	Standardized prevalence %	CMFL mf/ss
Batié	Varvateon	Mouhoun	306	236	4	1.69	1.8	0.03
	Yapoteon	Bambassou	315	245	1	0.41	0.4	0.02
	Ferkane	Mouhoun	199	134	1	0.75	0.6	0.01
	Ouadiel	Mouhoun	396	287	0	0	0	0
	Zindi	Mouhoun	428	289	2	0.69	0.8	0.01
	Tehini-Sud	Mouhoun	319	220	4	1.82	1.8	0.05
	Béboula	Bambassou	186	130	0	0	0	0
	Kankouèra	Bambassou	189	122	0	0	0	0
	Djongbal	Mouhoun	373	264	0	0	0	0
	Toussana	Mouhoun	336	254	9	3.54	4	0.1
	**TOTAL**		**3047**	**2181**	**21**	**0.96**		
Gaoua	Borikouladori	Mouhoun	287	205	0	0	0	0
	Boukéro	Mouhoun	254	162	1	0.62	0.9	0.01
	Maragnawan	Mouhoun	200	140	1	0.71	0	0
	Banipoulé	Mouhoun	157	101	0	0	0	0
	**TOTAL**		**898**	**608**	**2**	**0.33**		
Diébougou	Danko Tanzou	Bougouriba	380	200	0	0	0	0
	Balignar	Bougouriba	298	177	1	0.56	0.6	0
	ZOPAL/ Mouvielo	Bougouriba	220	148	0	0	0	0
	Sinkiro	Bougouriba	299	191	0	0	0	0
	Moulé	Bougouriba	216	156	0	0	0	0
	Limania Hameaux	Bougouriba	273	130	0	0	0	0
	Wapassi	Bougouriba	186	126	0	0	0	0
	Navrikpè	Bougouriba	291	190	0	0	0	0
	Nipodja	Bougouriba	176	109	0	0	0	0
	**TOTAL**		**2339**	**1427**	**1**	**0.07**		
Dano	Koumon	Bougouriba	552	328	0	0	0	0
	Korégnon	Bougouriba	435	303	0	0	0	0
	Baho 1 et 2	Mouhoun	346	248	0	0	0	0
	Bonko	Mouhoun	372	236	0	0	0	0
	Manoa	Mouhoun	331	185	0	0	0	0
	Zambo Hameau	Bougouriba	202	105	0	0	0	0
	**TOTAL**		**2238**	**1405**	**0**	**0**		

Of the 29 villages surveyed, 20 had crude prevalences of zero (Table 4). Nine villages had some microfilarial positive persons, but crude and standardized prevalences were all recorded as below 5%, with crude prevalences ranging from 0.41% (Yapoteon) to 3.54% (Toussana). Among the nine positive villages, six villages were in the Batié health district (60% of villages surveyed in Batié), two in the Gaoua health district (50%) and one in the Diébougou health district (11%). Dano health district had all villages presenting prevalences of zero (0%). It is notable that only a single person was microfilarial positive amongst all of the villages surveyed in the Bougouriba valley (and that person was 40 years old with no history of migration in the preceding ten years).

The community microfilarial load (CMFL) in positive villages varied between 0.01 microfilariae per skin snip in Ferkane, Zindi and Boukero and 0.1 microfilariae per skin snip in Toussana and were lower than 0.5 microfilariae per skin snip in all survey villages (Table 4).

### 2018 Survey—characteristics of positive individuals and migration survey

Of the 24 individuals found to be infected by *O*. *volvulus* in the 2018 survey (Table 4), 15 were male (62.5% of positives compared with 54.9% in the total sample). The average age was 47 years with extremes of 14 and 67 years. The highest number of infected individuals was in the Batié health district (21 individuals), followed by the Gaoua with (2 individuals) and Diébougou (1 individual). The migration survey showed that from the 24 infected people, 20 had a history of migration during the previous ten years (15 had stayed in Ghana, including the three persons in the age group <19 years old, and five in Côte d’Ivoire). The four remaining positive individuals were presumably autochthonous.

The age group of 50+ years recorded the highest number of infected with 13 individuals (see Table 5). Individual average loads varied from 0.5 to 18.5 mf/ss, with the average microfilarial load across all infected females and males being 2.11 and 4.13 mf/ss respectively. Age groups of 20–34 years and 5–19 years old harbored the highest loads (4.75 mf/ss and 4.0 mf/ss respectively) (Table 5). The three youngest mf-positive persons (constituting the 5–19 years old group) were 14, 18 and 19 years old, and were from three widely separate communities in Batié District (Téhini-Sud, Toussana and Maragnawan) and all had a history of migration to Ghana, with relatives resident in both countries.

**Table 5:** Microfilarial load for positive individuals by age-group and sex.

Variable	Total number of individuals examined	Number of positive individuals	Microfilarial load mfs/ss (standard error)
**By sex**			
Male	2548	15	4.13 (1.33)
Female	3073	9	2.11 (0.96)
**By age group**			
5–19*	2882	3	4.00 (3.00)
20–34	1014	2	4.75 (4.25)
35–49	902	6	1.75 (0.96)
50 +	823	13	3.77 (1.43)

*Note: These three mf positive persons were 14, 18 and 19 years old respectively.

## Discussion

### Endemicity of onchocerciasis and effectiveness of vector control and ivermectin

There is no question that the vector control operations carried out by OCP from 1975 had resulted in a dramatic and beneficial reduction in levels of onchocerciasis in the *Sud-Ouest* region by the time they were discontinued in 1989. Pre-control data and post-control data are not abundant [24,37,38], but two sentinel sites reported by Agoua et al. [37] from the Bougouriba valley had pre-treatment prevalences of 82% and 84% with CMFLs of 36 and 98 mf/ss respectively, which had dropped to 1% and 2% prevalence, and 0.03 and 0.03 mf/ss post-treatment. Vector infectivity rates (with head L3s indistinguishable from *O*. *volvulus*) in 1990 had dropped to 0.04% and 0.06% respectively amongst parous flies. At that time, the threshold considered safe for stopping treatments was that there should be no more than one infective fly per thousand parous flies (= 0.1%) [37]. It is unclear why there was subsequently a recrudescence of infections, but there were only six sentinel villages and two vector collection points in the Bougouriba valley where the recrudescence appeared, and the most likely explanation is that these sentinel sites were not representative of the situation throughout the whole of the *Sud-Ouest* region and there remained pockets of infection based upon unrecognized man-made vector breeding sites [24]. Even so, there was evidence at the time that vector control may have been stopped prematurely [39]. Vector infectivity was low, but skin-snip surveys in 1990 indicated communities with a range of prevalences up to around 7% [24].

It is also indisputable that the mass distribution of ivermectin from 1996 to 2013 brought the recrudescence under control. In 1994, newly infected children were found with an incidence of >1.8% in some villages. Even in the areas where in 1991 a low vector infectivity rate (0.6%) was found, an increase to 5.9% was observed in 1995. During the period 1996–2011 there were financial restraints so that it was impossible to carry out surveys to assess the impact of MDA, but in 2011 the proposed change from control to elimination made such a survey absolutely necessary, and it was found that the mean prevalence across all villages was 2.63% (range 0.0–9.7%), and CMFL varied from 0 to 0.25 microfilariae per biopsy. It seems that the situation had been stabilized after 1995 by the MDA, but there had been no obvious improvement and onchocerciasis was still a public health problem, and this was probably the result of poor geographic coverage [24]. In a multi-country study, it had been shown that CDTI produced better coverage than other methods of MDA (such as mobile teams and Community Based Treatment with Ivermectin) [40], and so the change of strategy from control to elimination was also accompanied by a change to biannual CDTI in 2013 after its successful introduction to control the recrudescence in *Cascades* region in 2011 [41].

There has been significant progress towards elimination during the period 2011 to 2018. In 2018 there was nobody found to be microfilarial positive below the age of 14 (compared with 17 individuals in 2011), and the % of villages which contained positives of any age dropped from 75% to 31%. Mean prevalence rates across all villages dropped from 2.6% (range 0.0–9.7%) in 2011 to 0.4% (range 0.0 to 3.5%), with the mean and maximum CMFL dropping from 0.07 and 0.25 respectively in 2011 to 0.01 and 0.1 in 2018. Of the villages surveyed in 2011 (20 villages) and 2018 (29 villages) only 18 were common to both surveys, but using data from these 18 villages (presented in Tables 2 and 4) the drop in prevalence between 2011 and 2019 was more or less identical to that found using the full data set (mean prevalence rates dropped from 2.9% in 2011 to 0.01%, with the mean CMFL dropping from 0.08 in 2011 to 0.01 in 2018).

The findings in the 2018 survey are very encouraging because they suggest that the region is progressing toward onchocerciasis elimination. A previous study assessing the effectiveness of the CDTI in the *Cascades* region, (where onchocerciasis had also been recrudescent), showed that the strategy could be applied effectively for control of onchocerciasis in Burkina Faso [41], and other studies in other parts of West Africa, East Africa and Ethiopia have also demonstrated successful interruption of onchocerciasis transmission by biannual CDTI [16,17,42,43,44,45]. Therefore, it seems probable that continuation of CDTI in the *Sud-Ouest* region of Burkina Faso, with rigorous supervision of the community distributors by health professionals, could quickly reach the goal of interruption of transmission. It remains uncertain how many more rounds of biannual CDTI might be necessary to achieve elimination of transmission, but WHO currently recommends a threshold of 0.1% threshold by OV-16 ELISA in children under 10 years, and our survey found 0% microfilarial skin-snip positive children under 14 years and 0.4% in all age groups. The precise difference in sensitivity between the two tests is unclear [33], so it would be unreliable to try to convert the skin-snip prevalence into OV-16 ELISA prevalence.

This study was designed with the objective of assessing the decline in the infection and not as a survey to decide if it was safe to stop mass drug administration (as defined by WHO) [34]. Therefore, the use of skin snips is justified because the skin-snip method is cheap, highly specific, familiar to both operatives and the public in Burkina Faso, and is directly comparable to historical data. Participation rate to skin snip diagnosis in the 2018 survey was found to be over 60% of the total population everywhere in the selected villages. There has been some concern that skin-snips are painful and this can lead to poor acceptance or refusal by communities during the epidemiological survey [16,46], but we did not find this to be a problem. It is possible that the awareness and communication campaign carried out by the local health professional and community leaders prior to the survey may have played an important role in the successful mobilization of population, along with the memory in the older people of the terrible situation caused by onchocerciasis in the past. Whatever the explanation, this study, and other studies in Burkina Faso [25,26], have generally found that the community participation rate has been satisfactory despite the long use of skin-snips in onchocerciasis epidemiological surveys.

However, even when communities accept the skin-snips, this is not a suitable diagnostic method to decide when to stop mass drug administration, because it has poor sensitivity when prevalence is low [33,34], and currently WHO recommends OV-16 tests on children using ELISA serology, along with O-150 pool-screening of vectors for the presence of *O*. *volvulus* DNA [34,47].

### 2018 migration survey

The migration survey which was part of the 2018 epidemiological survey was intended to help assess the possible origins of infections in microfilarial positive people. In this context it is notable that 83% of people who tested positive for microfilariae of *O*. *volvulus* had a history of cross-border travel (including the three youngest positives). Furthermore, Batié district recorded the most infected people, and this district that is closest to Ghana and is subject to significant cross-border human migration for several (usually economic) reasons. Some of people went to Ghana to work in plantations, and others for trading. It is possible that these people became infected in Ghana, but it is also possible that they became infected in Burkina Faso before CDTI was introduced. In either case, their history of migration may have resulted in missing rounds of ivermectin treatment, and this may be the most important issue with respect to effective CDTI and elimination of transmission. In any case, it is clear that the infected migrant people pose a risk for both Ghanaian and Burkinabè communities, especially because they included people with relatively high microfilarial loads. Cross-border collaboration between the countries concerned is necessary and urgent to ensure that these migrants are receiving doses of ivermectin in one country or the other so as to curb the threat they pose and to facilitate the progress towards elimination [47].

## Conclusion

Onchocerciasis was reduced to a very low level by historical vector control by OCP in the *Sud-Ouest* region of Burkina Faso, and the reasons for the resurgence after vector control had ceased are unclear, but were probably because the sentinel sites were not representative of the situation throughout the region as a whole (because of pockets of infection remained based on unrecognized, man-made vector breeding sites), and vector control was stopped prematurely. Subsequent MDA with ivermectin seems to have stabilized the recrudescence, and our study has shown that five years after the introduction of biannual CDTI onchocerciasis prevalence and intensity in the selected villages have been reduced, and were below 5% and 0.5 microfilariae/skin-snip (mfs/ss) respectively. CDTI with high coverage twice per year with enthusiastic and encouraging supervision of the community distributors by health professionals during campaign must continue to achieve an interruption of onchocerciasis transmission in the next few years. The historical resurgence of infections after vector control had been stopped is a warning of what might happen when the numbers of sentinel villages and vector collection sites are insufficient to cover an area, and it would be advisable to select yet more sentinel villages and sufficient numbers of vector collection sites to have confidence in any future epidemiological and entomological surveys.

## Supporting information

S1 Foreign Language Abstract French Language Title and Abstract. (PDF)

S1 Table Infection data from positive individuals during epidemiological surveys 2018. (PDF)

S2 Table Numbers of Mfs in positive people 2011 & 2018. (PDF)

S1 Fig Location of the vector breeding sites nearest to the 2018 survey sites in the four health districts in the Region Sud-Ouest of Burkina Faso. (PDF)
