# Supplementary material for: Progress towards elimination of onchocerciasis in the Region du Sud-Ouest of Burkina Faso which was previously subject to a recrudescence event after vector control
Source: PLoS Negl Trop Dis. 2024 Apr 29;18(4):e0012118. doi: 10.1371/journal.pntd.0012118 (PMC11057763; doi:10.1371/journal.pntd.0012118)
Supplement: S1 Foreign Language Abstract — (PDF) [file pntd.0012118.s001.pdf]

PLoSNTDs

Progress towards elimination of onchocerciasis in the *Region du Sud-Ouest* of Burkina Faso which was previously subject to a recrudescence event after vector control

By: Achille Sindimbasba Nikièma, Lassane Koala, Rory J. Post, Appolinaire Kima, Justin Compaoré, Claude M. Kafando, Jean Baptiste Nana, Clarisse Bougouma, Babacar Faye, Soungalo Traoré & Roch Kounbobr Dabiré

SUPPORTING INFORMATION

S1 Foreign Language Abstract:
French Language Title and Abstract (PDF)

Title:

Progrès vers l'élimination de l'onchocercose dans la Région du Sud-Ouest du Burkina Faso qui a connue une recrudescence après la lutte antivectorielle

Résumé:

Contexte: La région du Sud-Ouest du Burkina Faso (en particulier la vallée de la Bougouriba) a été historiquement problématique en ce qui concerne la lutte contre l'onchocercose, avec une recrudescence des infections après l'arrêt de la lutte antivectorielle menée dans le cadre du programme de lutte contre l'onchocercose de l'OMS en 1989. Après 1996, la distribution de masse à l'ivermectine a été exécutée pour contrôler la recrudescence de l'infection et et l'éliminer en tant que problème de santé publique. Cependant, en 2010, l'OMS a changé de paradigme, passant du contrôle à l'élimination de l'onchocercose, et en 2013, un traitement semestriel à l'ivermectine sous Directives Communautaires (TIDC) a été institué. Des enquêtes épidémiologiques ont été menées en 2011 et en 2018 pour déterminer si le TIDC entraînait une baisse des niveaux d'infection et des progrès vers l'élimination.

Méthodologie/principaux résultats: Une étude transversale a été menée dans 20 villages de quatre districts sanitaires en 2011 et dans 29 villages en 2018. Les personnes âgées de cinq ans et plus ont été examinées par biopsie cutanée exsangue, puis la prévalence et la charge microfilarienne ont été déterminées pour chaque village.
En 2011, 75% des villages avaient enregistré individus positifs et 20% avaient des prévalences >5%, avec une prévalence moyenne dans tous les villages de 2,63% (compris entre 0,0 - 9,7%), et une charge microfilarienne communautaire variant de 0 à 0,25 microfilaire par biopsie. En 2018, neuf villages (=31% du total) ont enregistré des individus positifs, avec des prévalences allant de 0,41% à 3,54%, et une prévalence moyenne dans tous les villages de 0,37%. La charge microfilarienne communautaire était comprise entre 0 et 0,1. Parmi les personnes positives à la microfilariose, 87% avaient des antécédents de migration.

Conclusions et signification: L'endémicité de l'onchocercose dans la région du Sud-Ouest a été réduite à des niveaux très bas et semble progresser vers l'élimination. Nos résultats ont indiqué que le Traitement à l'Ivermectine sous Directives Communautaires semestriel a un impact positif, mais qu'il devrait se poursuivre pendant un certain nombre d'années pour assurer l'élimination de la transmission. Cependant, les progrès vers l'élimination ont une histoire mouvementée dans cette région, et il serait souhaitable de sélectionner davantage de villages sentinelles pour plus de fiabilité dans les futures enquêtes épidémiologiques et entomologiques, en particulier les enquêtes Stop-MDA.
