# Supplementary material for: Progress towards elimination of onchocerciasis in the Region du Sud-Ouest of Burkina Faso which was previously subject to a recrudescence event after vector control
Source: PLoS Negl Trop Dis. 2024 Apr 29;18(4):e0012118. doi: 10.1371/journal.pntd.0012118 (PMC11057763; doi:10.1371/journal.pntd.0012118)
Supplement: S1 Table — (PDF) [file pntd.0012118.s002.pdf]

PLoSNTDs

Progress towards elimination of onchocerciasis in the *Region du Sud-Ouest* of Burkina Faso which was previously subject to a recrudescence event after vector control

By: Achille Sindimbasba Nikièma, Lassane Koala, Rory J. Post, Appolinaire Kima, Justin Compaoré, Claude M. Kafando, Jean Baptiste Nana, Clarisse Bougouma, Babacar Faye, Soungalo Traoré & Roch Kounbobr Dabiré

SUPPORTING INFORMATION

S1 Table:

Infection data from positive individuals during epidemiological survey 2018

Village	Person	Mfs left	Mfs right	Mean
Varvatan	1	1	0	0.5
Varvatan	2	3	0	1.5
Varvatan	3	2	0	1
Varvatan	4	2	1	1.5
Yapoteon	1	12	1	6.5
Tehini Sud	1	1	1	1
Tehini Sud	2	9	11	10
Tehini Sud	3	9	0	4.5
Tehini Sud	4	3	13	8
Toussana	1	35	2	18.5
Toussana	2	13	3	8
Toussana	3	2	0	1
Toussana	4	0	2	1
Toussana	5	1	2	1.5
Toussana	6	2	0	1
Toussana	7	3	15	9
Toussana	8	2	0	1
Toussana	9	1	0	0.5
Zindi	1	1	0	0.5
Zindi	2	3	1	2
Ferkane	1	1	0	0.5
Maragnawan	1	0	2	1
Boukero	1	1	0	0.5
Balignar	24	1	0	0.5
