# Supplementary material for: Progress towards elimination of onchocerciasis in the Region du Sud-Ouest of Burkina Faso which was previously subject to a recrudescence event after vector control
Source: PLoS Negl Trop Dis. 2024 Apr 29;18(4):e0012118. doi: 10.1371/journal.pntd.0012118 (PMC11057763; doi:10.1371/journal.pntd.0012118)
Supplement: S2 Table — (PDF) [file pntd.0012118.s003.pdf]

PLoSNTDs

Progress towards elimination of onchocerciasis in the *Region du Sud-Ouest* of Burkina Faso which was previously subject to a recrudescence event after vector control

By: Achille Sindimbasba Nikièma, Lassane Koala, Rory J. Post, Appolinaire Kima, Justin Compaoré, Claude M. Kafando, Jean Baptiste Nana, Clarisse Bougouma, Babacar Faye, Soungalo Traoré & Roch Kounbobr Dabiré

SUPPORTING INFORMATION
S2 Table: Numbers of Mfs in positive people 2011 & 2018

S2a Table:
Results of the 2011 skin-snip epidemiological survey showing numbers of microfilaria in positive people in all study villages.

	Village (number positive)	Positive people	Mfs Left	Mfs Right
**1**	Béboula (0)	0	-	-
**2**	Kankouèra (2)	1	2	0
		2	3	0
**3**	Téhini Sud (7)	1	1	25
		2	1	1
		3	2	1
		4	7	2
		5	2	1
		6	1	1
		7	2	1
**4**	Djonbal (27)	1	1	2
		2	1	0
		3	14	9
		4	17	45
		5	0	3
		6	2	13
		7	1	1
		8	1	0
		9	2	1
		10	17	22
		11	0	4
		12	1	0
		13	1	8

		14	1	0
		15	0	1
		16	1	0
		17	0	1
		18	9	3
		19	1	1
		20	1	1
		21	23	16
		22	2	0
		23	1	0
		24	1	6
		25	26	61
		26	32	58
		27	1	6
**5**	Toussana (27)	1	2	18
		2	1	1
		3	15	12
		4	0	1
		5	5	6
		6	5	8
		7	5	12
		8	0	1
		9	2	4
		10	2	1
		11	2	0
		12	12	15
		13	1	1
		14	22	25
		15	3	1
		16	0	2
		17	5	9
		18	22	25
		19	1	1
		20	1	0
		21	1	2
		22	27	10
		23	10	18
		24	0	2
		25	2	1
		26	6	2
		27	7	11
**6**	Ferkane (9)	1	1	6
		2	2	3
		3	6	3
		4	4	3
		5	8	6
		6	38	25

		7	12	17
		8	12	7
		9	1	0
**7**	Yapotéon (6)	1	0	1
		2	2	1
		3	1	0
		4	0	1
		5	2	0
		6	3	0
**8**	Zindi (3)	1	0	1
		2	1	1
		3	22	24
**9**	Boukéro	0	-	-
**10**	Maragnawa (5)	1	2	0
		2	22	2
		3	1	0
		4	1	0
		5	2	5
**11**	Navrikpe (0)	0	-	-
**12**	Nipodja (0)	0	-	-
**13**	Balignar (7)	1	1	1
		2	0	1
		3	4	2
		4	1	1
		5	0	2
		6	6	12
		7	2	3
**14**	Sikongo (1)	1	7	15
**15**	Wapassi (1)	1	48	29
**16**	Danko Tanzou (10)	1	1	0
		2	17	25
		3	6	10
		4	2	1
		5	0	1
		6	2	6
		7	1	0
		8	1	0
		9	2	25
		10	12	5
**17**	Benkadi (3)	1	0	2
		2	10	12
		3	1	3
**18**	Manoa (3)	1	1	1
		2	1	0
		3	16	2
**19**	Baho (1)	1	2	3
**20**	Korégnon (1)	1	1	0

S2b Table:
Results of the 2018 skin-snip epidemiological survey showing numbers of microfilaria in positive people in all study villages.

Village	Positive People	Mfs Left	Mfs Right
Varvateon (4)	1	1	0
	2	3	0
	3	2	0
	4	2	1
Yapoteon (1)	1	12	1
Ferkane (1)	1	1	0
Ouadiel (0)	0	-	-
Zindi (2)	1	1	0
	2	3	1
Tehini-Sud (4)	1	1	1
	2	9	11
	3	9	0
	4	3	13
Béboula (0)	0	-	-
Kankouèra (0)	0	-	-
Djongbal (0)	0	-	-
Toussana (9)	1	35	2
	2	13	3
	3	2	0
	4	0	0
	5	1	2
	6	2	0
	7	3	15
	8	2	0
	9	1	0
Borikouladori (0)	0	-	-
Boukéro (1)	1	1	0
Maragnawan (1)	1	0	2
Banipoulé (0)	0	-	-
Danko Tanzou (0)	0	-	-
Balignar (1)	1	1	0
ZOPAL/ Mouvielo (0)	0	-	-
Sinkiro (0)	0	-	-
Moulé (0)	0	-	-
Limania Hameaux (0)	0	-	-
Wapassi (0)	0	-	-

Navrikpè (0)	0	-	-
Nipodja (0)	0	-	-
Koumon (0)	0	-	-
Korégnon (0)	0	-	-
Baho 1 & 2 (0)	0	-	-
Bonko (0)	0	-	-
Manoa (0)	0	-	-
Zambo Hameau (0)	0	-	-
