# Supplementary material for: Progress towards elimination of onchocerciasis in the Region du Sud-Ouest of Burkina Faso which was previously subject to a recrudescence event after vector control
Source: PLoS Negl Trop Dis. 2024 Apr 29;18(4):e0012118. doi: 10.1371/journal.pntd.0012118 (PMC11057763; doi:10.1371/journal.pntd.0012118)
Supplement: S1 Fig — (PDF) [file pntd.0012118.s004.pdf]

PLoSNTDs

Progress towards elimination of onchocerciasis in the *Region du Sud-Ouest* of Burkina Faso which was previously subject to a recrudescence event after vector control

By: Achille Sindimbasba Nikièma, Lassane Koala, Rory J. Post, Appolinaire Kima, Justin Compaoré, Claude M. Kafando, Jean Baptiste Nana, Clarisse Bougouma, Babacar Faye, Soungalo Traoré & Roch Kounbobr Dabiré

SUPPORTING INFORMATION

S1 Figure:

Location of the vector breeding sites nearest to the 2018 survey sites in the four health districts in the Region Sud-Ouest of Burkina Faso.

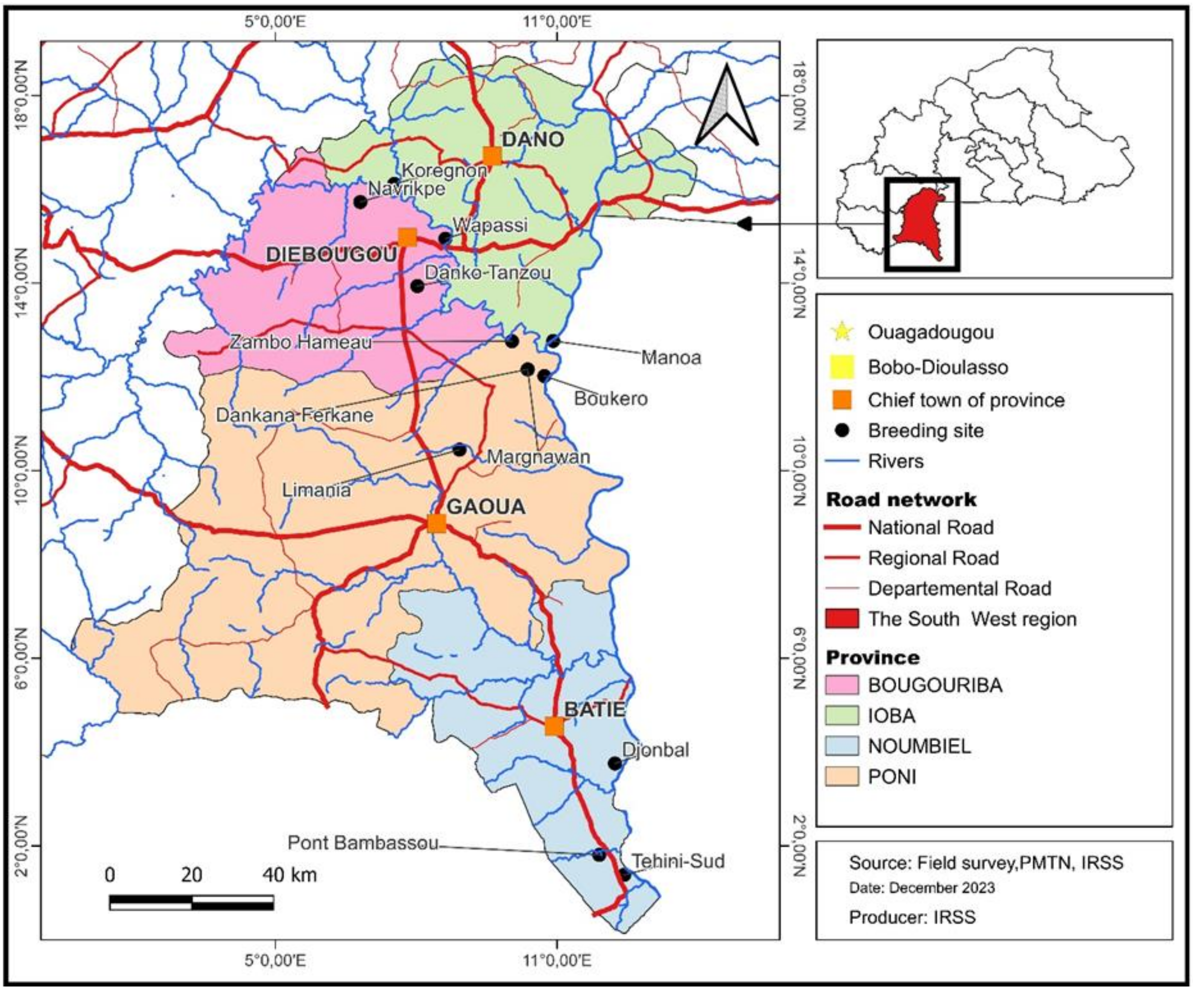

Note: S1 Figure was constructed by PMTN (*Programme national de lutte contre les Maladies Tropicales Négligées* which is part of the *Direction Générale de la Santé et de l'Hygiène Publique*) in conjunction with IRSS (*Institut de Recherche en Sciences de la Santé*). The basemap was obtained from the *Base Nationale des Données Topographique* (BNDT) of *l'Institut Géographique du Burkina* (IGB), and is provided freely to other government departments.
